# Unravelling insect declines: can space replace time?

**DOI:** 10.1098/rsbl.2021.0666

**Published:** 2022-04-20

**Authors:** Nico Blüthgen, Michael Staab, Rafael Achury, Wolfgang W. Weisser

**Affiliations:** ^1^ Ecological Networks Lab, Technische Universität Darmstadt, Schnittspahnstraße 3, 64287 Darmstadt, Germany; ^2^ Terrestrial Ecology, Department of Ecology and Ecosystem Management, Technical University of Munich, 85354 Freising, Germany

**Keywords:** arthropods, biodiversity loss, lag effects, land-use intensity, space-for-time substitution, time series

## Abstract

Temporal trends in insect numbers vary across studies and habitats, but drivers are poorly understood. Suitable long-term data are scant and biased, and interpretations of trends remain controversial. By contrast, there is substantial quantitative evidence for drivers of spatial variation. From observational and experimental studies, we have gained a profound understanding of *where* insect abundance and diversity is higher—and identified underlying environmental conditions, resource change and disturbances. We thus propose an increased consideration of spatial evidence in studying the causes of insect decline. This is because for most time series available today, the number of sites and thus statistical power strongly exceed the number of years studied. Comparisons across sites allow quantifying insect population risks, impacts of land use, habitat destruction, restoration or management, and stressors such as chemical and light pollution, pesticides, mowing or harvesting, climatic extremes or biological invasions. Notably, drivers may not have to change in intensity to have long-term effects on populations, e.g. annually repeated disturbances or mortality risks such as those arising from agricultural practices. Space-for-time substitution has been controversially debated. However, evidence from well-replicated spatial data can inform on urgent actions required to halt or reverse declines—to be implemented in space.

## 1. How spatial evidence can help

The term ‘insect decline’ typically refers to a *temporal* pattern, namely a significant downward trend of insect abundance or diversity over multiple years at a given location, or consistent negative interannual trends across locations. Variation in temporal trends is typically based on comparison in more than one location, making it also a spatial pattern. Can analyses across sites or regions, thus, reveal drivers of declines or increases? Might comparisons across locations *alone* suffice to draw inference about causes and to suggest mitigation strategies, even without long-term time series? Hence, does **space-for-time substitution** help to understand insect declines?

**Figure 1.**
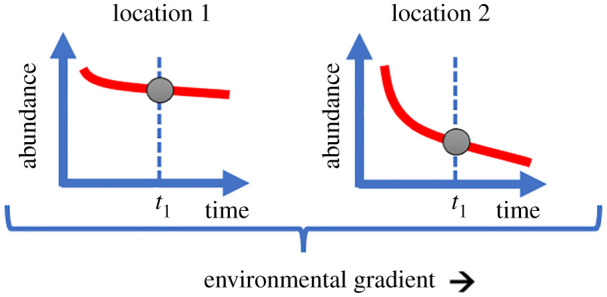
Schematic representation of two trends of insect abundance in two locations. Generally, environmental variation in space (across locations) can help to unravel the drivers of the temporal decline. The main argument in this paper is that spatial comparisons during a single year (*t*_1_) alone can already hold valuable information for drivers of declines when insights from long-term time series of insects and drivers are limited. This hypothesis assumes that populations were continuously affected by the environmental gradient over time (before *t*_1_) and had similar starting conditions or carrying capacity.

Here, we argue that analyses of potential drivers of declines and hazards, as well as mitigation strategies and conservation measures [1,2], should additionally make use of the substantial body of literature and evidence from studies across space, i.e. relationships with environmental conditions or land use across sites. The existing (and growing) knowledge on effects in space exceeds the potential for detectable drivers of temporal trends within reasonable time (figure 1). With some exceptions (e.g. [3]), the number of sites is usually higher than the number of years (e.g. [4,5]), hence spatial analyses hold a high statistical power. Observational, comparative studies across sites and gradients are an important approach in ecology, and a useful source of evidence for environmental drivers of variation in insect abundance and diversity. Moreover, controlled experimental treatments *in situ* or uncontrolled ‘natural experiments’ such as different land-use practices by farmers or other gradients in habitat disturbance, management and restoration [6,7] can be particularly informative for the impact of drivers. From these previous works, we know multiple environmental hazards and land-use impacts on insect communities, and it seems likely that such drivers of spatial trends are also relevant for temporal declines despite theoretical concerns for space-for-time substitution (see below).

## 2. Limitations of time series

Evidence about long-term trends of insects over several decades is scarce and poorly replicated [8], as it requires long-term monitoring with standardized methodology. Some historical, regional records of species occurrences are available [9–11], partly involving community scientists [4,12], but are strongly biased towards Europe and North America [13–15].

In order to search for *drivers* of the decline, we typically focus on the interaction term between time and environment (*T* × *E*) or compare different independent scenarios, e.g. habitats or regions (figure 1). However, until the temporal resolution improves in the future, we could also focus on *E* alone to assess the relative contribution of different drivers, irrespective of their contribution to *T* × *E*. An evaluation of potential drivers and actions to promote favourable environmental conditions in space may also prove useful regardless of whether it is a major driver of the temporal decline (*T* × *E*).

Stimulated by the debate on—and relevance of—insect declines, many monitoring campaigns have started recently and will reveal important insights into insect trends in the decades to come. By contrast, short time series lack the statistical power to detect real temporal trends ([16], see also [17] versus [18]). Similarly, a recent controversy about appropriate measures for diversity losses such as the ‘Living Planet Index’ highlights the sensitivity of limited time-series trends to outliers, bias and inclusion criteria (e.g. [19,20]). Sparked by such concerns, several studies have even challenged the existence of insect declines. For example, a prominent study [21] has questioned declines in monitoring data across several Long Term Ecological Research Network (LTER) sites in the United States, but suffered from statistical flaws [22] and ignored a concomitant increase of sampling intensity, which led to spurious conclusions [23]. Appropriate interpretations of temporal trends require a sound knowledge of environmental co-variables—in both space and time. Agricultural intensification, for example, goes along with larger field sizes, fewer buffer strips and hedges, reduced crop diversity and increased application of pesticides and fertilizers [15]. These and several other covarying environmental factors may be plausible drivers, but have not been commonly quantified over time with existing insect monitoring schemes. Scientific debates on trends and their causes are therefore ongoing [24,25]. When the evidence for declines is mixed or weak, a search for underlying mechanisms and recommendations for action remains even more challenging [15,26]. Critically, considering that insect communities have been studied intensively, one question arises: do we really understand so little?

Irrespective of the lengths of time series, **variation of trends (slopes) across space** is pronounced and partly obscures the detection of regional average or even global trends. This variation likely corresponds to variation in the multiple drivers that act in parallel or interact in complex ways. In a global meta-analysis, negative trends prevailed in terrestrial ecosystems while positive trends were found in freshwater ecosystems [14]. Like other meta-analyses (see [16]), this study needed to compare trends of different quality and time length; hence data inclusion criteria and conclusions were subject to controversies [27,28]. Despite such challenges, it is key to understand the drivers of variation in trends in space to unravel the multifactorial causes of a decline in time. Importantly, spatial variation is meaningful—and has the potential to shift the focus on mere statistical detectability of insect declines to an improved understanding of the drivers underlying the evident losses in individual ecosystems and locations [16].

## 3. Evidence for spatial drivers

In general terms, negative population growth can occur through low birth rates (e.g. mediated through resource limitation or changes in environmental conditions) or through high mortality rates (e.g. due to disturbances and hazards such as pesticides, mowing, ploughing, pollution, pathogens and predation), or both. Many disturbances, including agricultural practices, are continuous, repeated annually and have additive effects. While disturbance rates may change over time (e.g. by increasing mowing frequency or pesticide application), it is important to note that their repetition (or continuation) alone at the same level may qualify as a driver of insect declines. Even immediate but discontinued impacts may contribute to more long-lasting declines by ‘lag effects’ of reduced population sizes or genetic disadvantages of small populations. For the future, it will be important to disentangle and understand resource- versus disturbance-mediated effects, immediate or lag effects, reversible and long-term drivers on insect populations and communities (see electronic supplementary material, Distinguishing continuous drivers and single events, immediate and lag responses).

**Figure 2.**
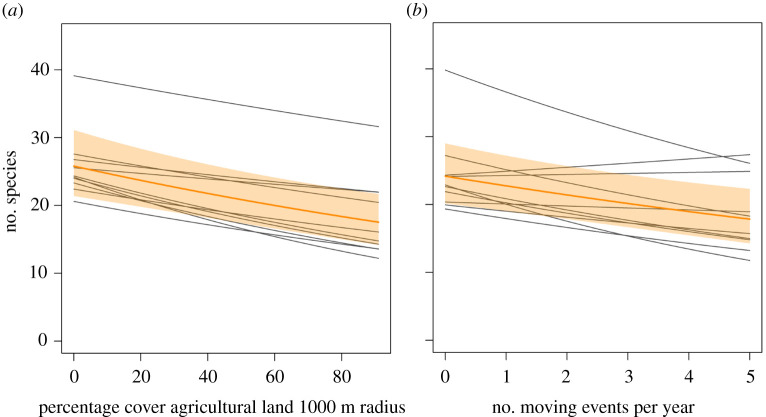
Example for a driver in space that mirrors a driver in time, illustrated with data on arthropod species richness of 150 German grasslands sampled annually from 2008 to 2017. (*a*) Sites surrounded by more agricultural land had over all years a lower number of species (marginal prediction of a Poisson mixed-effects model: orange line with 95% CI as shaded polygon); in space, the relationship between cover of agricultural land and species richness was negative in every individual year (grey lines). (*b*) Arthropod species numbers are lower in sites that are mown more often per year and this relationship was prevalent in all but two individual years (grey lines). Details on data and analyses are available in the electronic supplementary material.

Several potential drivers of temporal declines are clearly detectable in data across space, e.g. when comparing different land-use categories or intensity levels. For example, along a gradient of land-use intensity in the 150 grasslands within the ‘Biodiversity Exploratories' project [29], a recent study with a 10-year time series showed that the proportion of area used as agricultural land around the sampling sites was a strong predictor (time × agricultural land interaction) of temporal declines in arthropod biomass, abundance and species richness [5]. Grasslands surrounded by more agricultural land had stronger declines over time. Note that the amount of agricultural land in a Central European landscape is a static, not a dynamic variable within the considered time frame of one decade. The corresponding relationship is also evident in space (*E*), with a negative relationship between the proportion of agricultural land and insect species richness in each year of the time series (figure 2*a*). Thus, in this case, we can identify important risks and potential drivers from focusing on space alone within few years or a single year only, without the need of compiling long-term data.

In the same 150 grasslands, we also found significant *spatial* declines with land-use intensity in 52% of the grasshopper [30], 34% of the cicada [31], 28% of the moth [32] and 19% of the bee and wasp species [33]. Rather than over time, these trends were analysed in a single year by comparing species’ abundances along the land-use gradient. Variation in abundance across sites was strongly related to mowing intensity; individual grasslands were mown from zero (pastures) to five times per year. Grazing by cows, sheep or horses had comparably little or no negative effect; hence meadows had fewer insects than (unmown, but grazed) pastures. The 10-year time series confirms the negative effects of mowing intensity reported for grasshoppers, cicadas, moths and hymenopterans: there was an overall negative relationship between mowing and arthropod species richness. The negative spatial trend occurred across almost all years (figure 2*b*); hence, it can be often concluded from comparisons within a single year. Such strong impacts of mowing are expected: mowing represents a disturbance with immediate changes in the structure and microclimate of the vegetation layer, and particularly a severe mortality risk for animals. Most insects and spiders face very high losses during mowing and subsequent processing, with mortality rates typically over 50–80% resulting from modern machinery and low cutting height applied in meadows [34,35] or along road verges [36]. Mowing is repeated every year and can thus represent a driver of long-term declines irrespective of temporal trends in land-use intensity. Mortalities are, furthermore, additive with every mowing event within a year [30]. Mowing by modern machinery—much more than grazing—thus represents a substantial sink for insect populations that live in cultivated grasslands and lawns, or in huge areas provided by road or field margins, either permanently or at least as part of their life cycle [36]. Moreover, it exerts an extrinsic density-independent mortality risk, unlike bottom-up regulation by resource limitation or top-down regulation through predation or pathogens. The high mortalities in a mown area are often masked by subsequent recolonization by insects from (unmown) surroundings [30], but a mere redistribution of surviving individuals does not compensate for overall losses in populations.

Both mowing and grazing prevent the growth of shrubs and trees, and increase the habitat suitability for grassland plants and also for insects that require particular conditions including warmer microclimates [37]. However, grazing clearly represents a preferable option to avoid the immediate large-scale mortality of insects. Similar to losses through mowing, other drivers of insect decline (e.g. pollution, pesticides, fragmentation) can be systematically studied across space (table 1).

**Table 1.** Analysing potential drivers of insect decline using time series (left column) or variation across sites (spatial approach, right column). Drivers may cause immediate responses of insect populations and/or lag effects and may vary in quantity or quality, e.g. pesticide application may become more frequent, more effective or both. Note that many drivers represent continuous or regular disturbances and are repeated annually, e.g. those related to agricultural practice, so they do not necessarily have to increase to trigger long-term declines. The list is just exemplary rather than complete, and only a single reference is given as an example for each driver.

time series	spatial approach
**(1) *local, site-specific drivers***	
*habitat change and land-use intensification*	
long-term variation in habitat quality or land-use intensification	gradients of habitat quality or land-use intensity [30]
*change in habitat area and isolation*	
long-term trajectories of changes in habitat composition	comparing habitat islands of varying sizes, shapes and degrees of isolation [38]
*urbanization, sealing*	
trajectories starting before urbanization	comparisons along urbanization gradients [39]
*mowing* (frequency, timing, speed, machine impact and cutting height)	
time series between years or short term before/after mowing [34]	variation in mowing regime across meadows [30]
*pesticides*	
change in application frequency or toxicity [40]	different farms or application treatments [41], controlled experiments
*veterinary medicine* (parasiticides, antibiotics)	
change in application practice over time [42]	comparing different farms [42]
*chemical pollution*	
change in pollution level or quality	comparing pollution levels, e.g. heavy metal gradient [43]
*light pollution*	
change in light pollution over time [44]	comparing illuminated versus dark sites [45]
*eutrophication and fertilization*	
long-term change in nutrient stocks	nutrient variation, e.g. soil N gradient [46]
*availability of resources, hosts or mutualistic partners*	
temporal trends of resources or host plants	spatial variation in resource or host plant abundance [47]
*fire impacts*	
increase in fire impacts, trajectories before and after fire events [48]	comparing control with burning treatments [49]
*traffic, car strikes*	
change in traffic or car strikes	comparing roads with low traffic versus high traffic [50]
*restoration*	
long-term changes with restoration practice	comparing restored and unrestored sites [51]
**(2) *regional, landscape or global drivers***	
*fragmentation, expansion of agricultural areas* at the cost of (semi-)natural areas	
long-term trajectories of fragmentation	fragmentation gradients across the landscape [52]
*temperature or drought*	
long-term trends along with climatic data [40]	climatic gradients, e.g. elevation [53]
*biological invasions*	
trajectories before and after invasions [54]	variation in invader abundance [55]

## 4. Caveats against space-for-time substitution

We have argued that drivers of spatial variation in insect abundance, diversity and composition can be similar to those expected for temporal changes (figure 1). However, at least in theory, space is only an imperfect substitute for time for predictions of population or community responses, a concern that has also been raised for insect declines [8]. Fundamental assumptions of space-for-time substitution have been controversially debated, particularly in the context of diversity–climate relationships uncovered in fossil pollen profiles [56,57], current trajectories of vegetation succession [58,59] and predictions of climate envelope models [60]. Evidence for the validity of the underlying assumptions was mixed, ranging from conceptual [60,61] and empirical support [56,58,62,63] to strong reservations and critique [57,64,65]. For instance, re-population responses of a fish species to drought events were even better predicted by spatial than by temporal analyses [62], whereas spatial and temporal relationships between bird communities and landscape composition [64] and annual temperature [66] were not congruent.

Species communities are dynamic and undergo intrinsic and extrinsic variation, e.g. species co-occurrences and competition at different sites vary owing to random environmental events or legacies. Assuming static processes, and thus ignoring the legacy or dynamics at the site level, may thus bias the conclusions drawn from space-for-time substitution [58,65]. On the other hand, time scales of recognizable biodiversity loss, particularly insect declines, in the Anthropocene are relatively short. Relevant trends occur in one or few decades (e.g. [4,5]). Hence, methodological concerns about space-for-time substitution from the viewpoint of very long-term scales may be less severe [65] and local adaptations negligible [62]. Moreover, many insects are highly mobile and relatively good dispersers; hence they may rapidly respond to environmental variation in space, display compensatory dynamics and could be less affected by unknown variation in site history than organisms with poor dispersal. High dispersal capabilities and inter-site connectivity, and thus low beta diversity, represent feasible conditions for space-for-time substitution [62,63].

As a note of caution, spatial comparisons suffer from the fact that many potential drivers of insect decline are correlated and cannot be easily disentangled. Several confounding factors, e.g. different species pools, may additionally hamper a straightforward interpretation of spatial drivers. Uncontrolled confounding factors may, however, also affect time-series analyses, e.g. when some sites but not others include post-disturbance recovery [16]. Correlation is not causation, thus both spatial and temporal analyses should ideally account for covariates or even experimental treatments *in situ* at a relevant (spatial) scale. Nevertheless, there is substantial potential to detect relevant drivers across space [67], and the statistical power for tests across independent sites—including appropriate covariates—can be larger than for a limited number of time series. Spatial comparisons thus offer a promising, fast and often underestimated tool to understand drivers of biodiversity change.

## 5. Plea for monitoring and solutions in space

Sparked by the debate on insect decline, ecological research now intensifies its activities towards time-series data—and there are many good scientific reasons for doing so. It may appear that concurrent funding and publication models often doom spatial ‘monitoring’ of communities or basic comparisons across different types of habitats as unexciting ‘descriptive’ science. For successfully transferring relevant ecological knowledge into applications, however, we should overcome our attitude towards seemingly less ‘novel’ or ‘interesting’ science.

It should be emphasized that *both* temporal and spatial attempts can represent relevant contributions to understand and mitigate diversity losses. Ideally, sparse time-series data are complemented with spatial analyses and *in situ* experiments, which would be a promising combination of approaches [58,63,65]. Evaluations of different environmental effects on species communities, or simple experiments under field conditions, even when replicated from just a different region compared with previous work, can be highly relevant for conservation. More generally, the environmental and biodiversity crisis will require solution-based research, not only debates on scientific problems or statistical evidence. Global or regional temporal trends may or may not be reversible, which requires a more solid understanding of the drivers and their long-term effects. However, practical solutions at short-time horizons require action in specific areas, e.g. in preserving or restoring important habitats, improving corridors or changing land-use regimes. Hence, while the problem may be temporal, solutions are often inherently spatial.

## Data Availability

The source data underlying figure 2 are publicly available via the BExIS repository (https://doi.org/10.17616/R32P9Q) at https://www.bexis.uni-jena.de/PublicData/PublicDataSet.aspx?DatasetId=31182.
